# Image Recommendation System Based on Environmental and Human Face Information

**DOI:** 10.3390/s23115304

**Published:** 2023-06-02

**Authors:** Hye-min Won, Yong Seok Heo, Nojun Kwak

**Affiliations:** 1 Department of Electrical and Computer Engineering, Ajou University, Suwon-si 16499, Republic of Korea; dnjsgpals@ajou.ac.kr (H.-m.W.); ysheo@ajou.ac.kr (Y.S.H.); 2 Graduate School of Convergence Science and Technology, RICS, Seoul National University, Seoul 08826, Republic of Korea

**Keywords:** recommendation system, image recommendation system, human face, emotion recognition, HCI

## Abstract

With the advancement of computer hardware and communication technologies, deep learning technology has made significant progress, enabling the development of systems that can accurately estimate human emotions. Factors such as facial expressions, gender, age, and the environment influence human emotions, making it crucial to understand and capture these intricate factors. Our system aims to recommend personalized images by accurately estimating human emotions, age, and gender in real time. The primary objective of our system is to enhance user experiences by recommending images that align with their current emotional state and characteristics. To achieve this, our system collects environmental information, including weather conditions and user-specific environment data through APIs and smartphone sensors. Additionally, we employ deep learning algorithms for real-time classification of eight types of facial expressions, age, and gender. By combining this facial information with the environmental data, we categorize the user’s current situation into positive, neutral, and negative stages. Based on this categorization, our system recommends natural landscape images that are colorized using Generative Adversarial Networks (GANs). These recommendations are personalized to match the user’s current emotional state and preferences, providing a more engaging and tailored experience. Through rigorous testing and user evaluations, we assessed the effectiveness and user-friendliness of our system. Users expressed satisfaction with the system’s ability to generate appropriate images based on the surrounding environment, emotional state, and demographic factors such as age and gender. The visual output of our system significantly impacted users’ emotional responses, resulting in a positive mood change for most users. Moreover, the system’s scalability was positively received, with users acknowledging its potential benefits when installed outdoors and expressing a willingness to continue using it. Compared to other recommender systems, our integration of age, gender, and weather information provides personalized recommendations, contextual relevance, increased engagement, and a deeper understanding of user preferences, thereby enhancing the overall user experience. The system’s ability to comprehend and capture intricate factors that influence human emotions holds promise in various domains, including human–computer interaction, psychology, and social sciences.

## 1. Introduction

In today’s era where personalization is increasingly important, this research aims to provide tailored and personalized emotional services to users, positively impacting their daily lives. In modern society, we expect a high level of personalization and want to be managed as independent individuals. The importance of personalization has been emphasized in recent years due to the rise in single-person households and advancements in information technology. In this context, individual autonomy and independence are highly valued, and many people strive to manage their lives freely. Therefore, it is necessary to digitize emotional information and utilize technology that can automatically recognize human emotions and process emotional information according to the user’s situation, in order to provide customized emotional services in daily-life products or services. This technology can optimize the service provided by recognizing the user’s emotions and providing them with personalized emotional services, which are expected to have a positive impact on the user’s life. To meet these expectations, this paper proposes a novel system that goes beyond traditional recommender systems by incorporating real-time environmental information and offering tailored recommendations based on users’ emotional states.

A recommender system is a system that analyzes user preferences and interaction records to recommend personalized items [1,2]. There are various types of recommender systems, including content-based filtering, collaborative filtering, and hybrid recommender systems. Content-based filtering recommends items that have similar characteristics to those preferred by the user, relying on the features of items and providing recommendations even without explicit user preference information. Collaborative filtering, on the other hand, recommends items based on the preferences of other users, calculating the similarity of items that have been rated by users and recommending items that are similar to those preferred by other users. Collaborative filtering requires user preference information gathered from other users. Hybrid recommender systems combine the strengths of both content-based filtering and collaborative filtering, providing more accurate and personalized recommendations by considering both user preferences and item characteristics.

The proposed system differs from existing recommender systems by incorporating real-time environmental information, facial expression recognition, gender recognition, and age estimation. Furthermore, we implemented a hybrid recommender system that combines the strengths of content-based filtering and collaborative filtering techniques, ensuring a more precise matching of user preferences with recommended content. By considering both user preferences and item characteristics, the system ensures a more precise matching of user preferences with recommended content. However, what truly sets our system apart is the integration of real-time environmental information, facial expression recognition, gender recognition, and age estimation. By incorporating real-time environmental data, our system adapts to the user’s immediate context, taking into account factors such as weather conditions, lighting, and noise levels. This contextual information allows the system to recommend images that align with the user’s surroundings, enhancing the overall experience and mood. For instance, on a sunny day, the system can suggest bright and cheerful images, while on a rainy day, it can provide calming or cozy visuals. Furthermore, the system analyzes the user’s facial expressions, gender, and age in real-time to gain a deeper understanding of their emotional state and individual characteristics. This information is then utilized to generate personalized recommendations tailored to the user’s preferences and emotional needs. By considering these multiple dimensions, our system provides a holistic approach to personalized emotional services. The significance of this research lies in its ability to positively impact users’ daily lives by delivering customized emotional experiences. Tailored recommendations can uplift users’ moods, reduce stress, and improve overall well-being. The integration of real-time environmental information and individual characteristics not only enhances the system’s accuracy but also creates a more engaging and immersive user experience.

Merely providing feedback based on facial expression recognition is insufficient for delivering appropriate information to individuals. Therefore, feedback through emotional recognition necessitates the consideration of various factors. We have developed a system that provides suitable image information based on the user’s emotional state, considering environmental information, age, gender, and other relevant factors. To do this, we estimated real-time age and gender information from the user’s face and collected 20 points of environmental information. In our system, environmental information and user facial information are categorized into three stages based on principles from color psychology and environmental psychology. The outcomes are then input into a generation model to generate appropriate images. For estimation and recognition, we employ the Convolutional Neural Network (CNN) algorithm as the classifier and utilize the VGG16 model architecture. The CK+ dataset was used for facial expression recognition, and the MORPH dataset was used for age and gender estimation as training data. Environmental information is gathered using smartphone sensors, Really Simple Syndication (RSS) from the Korea Meteorological Administration, and OpenWeatherMap data. We estimate and recognize the user’s age, gender, and facial expression in real-time from the user’s image information captured through the camera.

The evaluation of the system was conducted through user studies. Participants evaluated the provided images in real-life scenarios and provided feedback. Through this process, we evaluated the system’s performance and user satisfaction, and identified areas for improvement. The results of the user study confirmed that the proposed system accurately identifies the user’s emotional state and preferences to provide personalized recommendations. Additionally, the image recommendations considering real-time environmental information had a positive impact on improving the user’s experience and mood. These findings validate the effectiveness of the proposed system in providing personalized emotional services. Therefore, these research results are considered an important contribution to the advancement of next-generation systems in the field of personalized emotional services.

In Section 2, Related Works, we review prior research in the fields of human–computer interaction and recommender systems, with a particular focus on relevant papers pertaining to recommender systems. Section 3, Human Psychology in Relation to the Environment, explores the impact of various environmental factors on human psychology, including high temperature, discomfort index, noise, and outdoor and indoor lighting. Section 4, Algorithms Used and Proposed Methods, includes subsections on Environmental Information (describing 15 weather-related data points obtained through public APIs from the Korea Meteorological Administration and OpenWeatherMap, five data points collected through sensors on smartphones) and Facial Expression Recognition and Age & Gender Estimation (introducing the techniques employed for these tasks). Section 5, Image Recommendation System, presents an image recommendation system that combines principles from color psychology, environmental data, and GAN algorithms to generate personalized images considering an individual’s emotional state and surrounding environment. Finally, to evaluate the system’s performance and effectiveness, Section 6 conducts a user satisfaction survey, consisting of user testing and surveys, to assess the performance of the personalized image recommendation system and gather feedback on user satisfaction.

## 2. Related Works

In the fourth industrial age, there is a growing emphasis on the importance of human factors (HF) (or ergonomics, HF/E). In 2000, the International Ergonomics Association (IEA) defined HF/E as a field of science concerned with understanding the interactions between humans and other elements of a system, and the profession of applying theory, principles, data, and methods to design to optimize human well-being and overall system performance [3]. HF/E research is divided into three main categories: physical ergonomics, cognitive ergonomics, and organizational ergonomics. Physical ergonomics studies loads and responses in the physical and physiological aspects of humans, including material handling, workplace layout, physical safety and health, and planning and repetition of work sequences. Cognitive ergonomics studies the effects of human interaction with other systems, including perception, memory, reasoning, motor response, and human–computer interaction. Organizational ergonomics studies the optimization and efficiency of sociotechnical systems, including organizational structures, policies, and processes.

This paper is grounded in the field of cognitive ergonomics, which focuses on the characteristics of human psychological activity and mental processes, and investigates their impact on the interaction between humans and systems [4]. Cognitive ergonomics explores various aspects such as perception, memory, reasoning, motor response, and human–computer interaction. Specifically, we classify human emotions based on user (face) and environmental (weather) information and generate new images through a deep generative model to establish a system that visually satisfies the user and stimulates emotions. This work contributes to the growing body of research in the field of HF/E, specifically in the area of Cognitive Ergonomics and human–computer interaction.

A recommendation system is a personalized method for discovering products or services that a user may be interested in [5]. The input to a recommendation system is based on user profile settings, item features, past interactions of the user with the system, and additional information such as time and location data. Recommendation systems can be classified into three types based on the type of input data: collaborative filtering, content-based filtering, and hybrid recommendation systems [6]. Collaborative filtering systems recommend items to users based on the preferences and behaviors of similar users, while content-based filtering systems recommend items to users based on the features of the items they have interacted with in the past. Hybrid recommendation systems combine the approaches of collaborative filtering and content-based filtering to provide more accurate and diverse recommendations to users. Recommendation systems have become increasingly important in various industries such as e-commerce, entertainment, and social media, as they can enhance user satisfaction, increase user engagement, and drive business revenue. However, developing effective recommendation systems still remains a challenging research problem due to issues such as data sparsity, cold start, and scalability.

Emotion recognition-based video recommendation systems have been proposed by several studies using machine learning and deep learning techniques to understand users’ emotions and interests. These systems use facial recognition and emotional recognition to recommend videos or images that can change users’ moods. Bokhare et al. proposed a video recommendation system that combines facial recognition and emotion recognition to recommend suitable videos based on users’ emotional states, using machine learning and deep learning algorithms [7]. The system can dynamically adjust according to users’ emotional states and offer personalized recommendations based on their viewing history and feedback. Choi et al. proposed a new recommendation method that utilizes users’ facial expression changes as a dynamic user profile, which addresses the new user problem and outperforms other systems in recommending to existing users [8]. Babanne et al. proposed a Personalized Video Recommendation (PVR) system that provides personalized entertainment recommendations based on users’ emotions, age, and gender [9]. The system utilizes face detection, feature extraction, and emotion detection algorithms to recommend videos based on their relevance to the target emotion, popularity among other users, and video categories.

## 3. Human Psychology According to Environment

The environment, including weather, temperature, humidity, and other factors, can have a significant impact on human psychology. In particular, high temperatures, discomfort indices, noise, and other factors can have negative effects on human psychology and health.

According to several studies, it has been revealed that high temperatures have a negative impact on both physical and mental health [10]. One of the key findings of the research is the potential of high temperatures to increase aggression and violence [10,11]. Adverse effects on mental health manifest as a rise in emergency room visits for mental disorders, incidents of suicides, and a decline in mental well-being [12]. Moreover, extremely high or low temperatures are associated with poor health outcomes. High temperatures are linked to heightened discomfort, frustration, impulsivity, and aggression among individuals [11]. Consequently, it is crucial to implement measures to mitigate the adverse impacts of high temperatures, especially in work environments where individuals spend significant amounts of time.

The Discomfort Index (DI) is a weather index that calculates the perceived level of heat discomfort, which has a significant impact on daily life and health, based solely on temperature and humidity [13,14]. The DI has considerable implications for human health. In regions with high DI, symptoms such as heatstroke and dehydration may manifest [15]. Higher DI values are associated with an elevated risk of heat exhaustion, heat cramps, and heatstroke [16]. Heatstroke is a life-threatening condition that can cause damage to the brain and other vital organs. Moreover, increased DI is linked to dehydration and other heat-related illnesses. Higher DI values can significantly impact the human body, leading to respiratory and bronchial dysfunction, increased discomfort, and a higher occurrence of negative phenomena such as higher crime rates, increased traffic accidents, and decreased work efficiency [17]. The following are responses based on the DI (%):

- 100∼80: Very high discomfort level, where all people feel unpleasantly hot.
- 75∼80: High discomfort level, where most people feel unpleasantly hot.
- 68∼75: Moderate discomfort level, where discomfort begins to set in.
- 0∼68: Low discomfort level, where all people feel comfortable temperatures.

**Figure 1:**
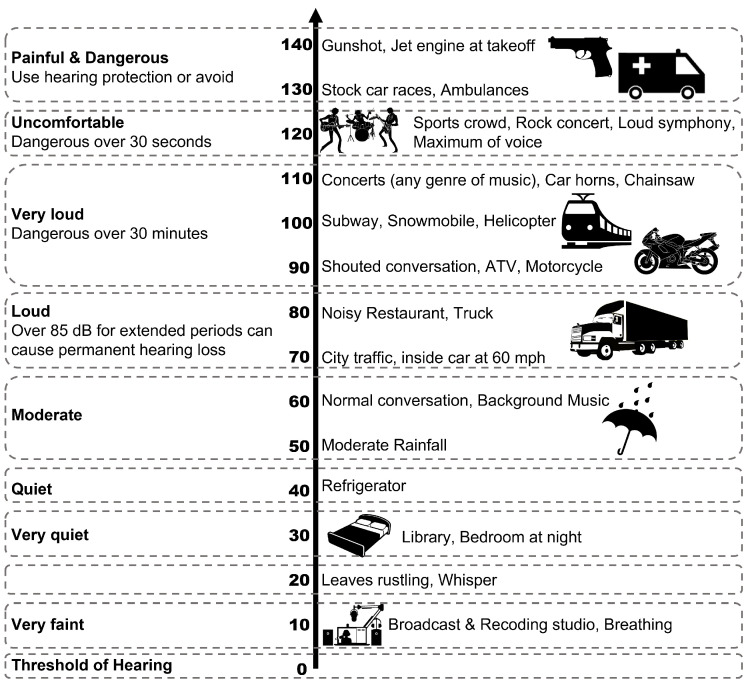
Noise levels.

Noise can be considered a negative environmental factor when it is unwanted or unnecessary [18]. Noise levels are depicted in Figure 1.

In residential areas, noise levels of 45 dB or less are appropriate, and exposure to sounds above 90 dB for an extended period can lead to hearing impairment. The negative effects of noise are influenced by the intensity, predictability, and control of the noise. Noise can affect human hearing, physical and mental health, work, and social behavior. The following are responses to noise:

- Reduced attractiveness: Noise at around 80 dB causes interpersonal relationships to deteriorate, as the level of friendliness towards others decreases due to the noise.
- Increased aggression: The presence of noise (at 60 dB) can lead to an increase in aggressive behavior compared to its absence.
- Decreased helpfulness: As noise levels increase, the frequency of people helping others decreases.
- Physical and Mental Health Disorders: Long-term exposure to noise (e.g., high-pitched sounds) can cause headaches, motion sickness, instability, arguments, anxiety, and impotence.

The outdoor lighting level is approximately 10,000 lux on a clear day. However, for indoor spaces, the recommended standard light levels by ISO vary depending on the type of activity performed [19,20]. Table 1 shows that higher illuminance is recommended for spaces where activities requiring high cognitive load and concentration, such as sewing and studying, are performed. On the other hand, spaces where activities with lower cognitive load and concentration, such as bedrooms, are performed, are recommended to have lower light levels.

## 4. Algorithm Used and Proposed Methods

### 4.1. Environmental Information

In this paper, external environmental information (weather) and surrounding environmental information were collected through websites and smartphone sensors.

**Figure 2:**
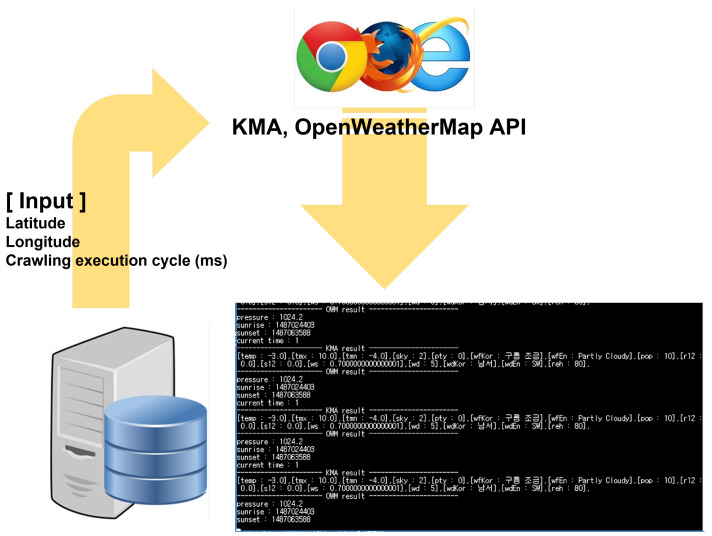
15 environment information values output: (1) KMA result: temperature, daily maximum temperature, daily minimum temperature, sky status, precipitation, weather (Korean, English), probability of precipitation, 12 hours expected rainfall, 12 hours expected snowfall, wind speed, wind direction (number, Korean, English), humidity, (2) OWM result: atmospheric pressure, sunrise time, sunset time.

The public API of the Korea Meteorological Administration and the OpenWeatherMap API were utilized to obtain 15 outdoor environmental information. These APIs can be executed in environments that support Visual Studio or the C++ language. When the latitude, longitude, and crawling execution cycle (in milliseconds) are provided as inputs to the API, the program outputs the corresponding environmental information values. However, in some places such as city outskirts, environmental information may not be collected. A program was developed in which environmental information is periodically received by sending the latitude and longitude of the experimental site and the crawling cycle to the API (Figure 2).

**Figure 3:**
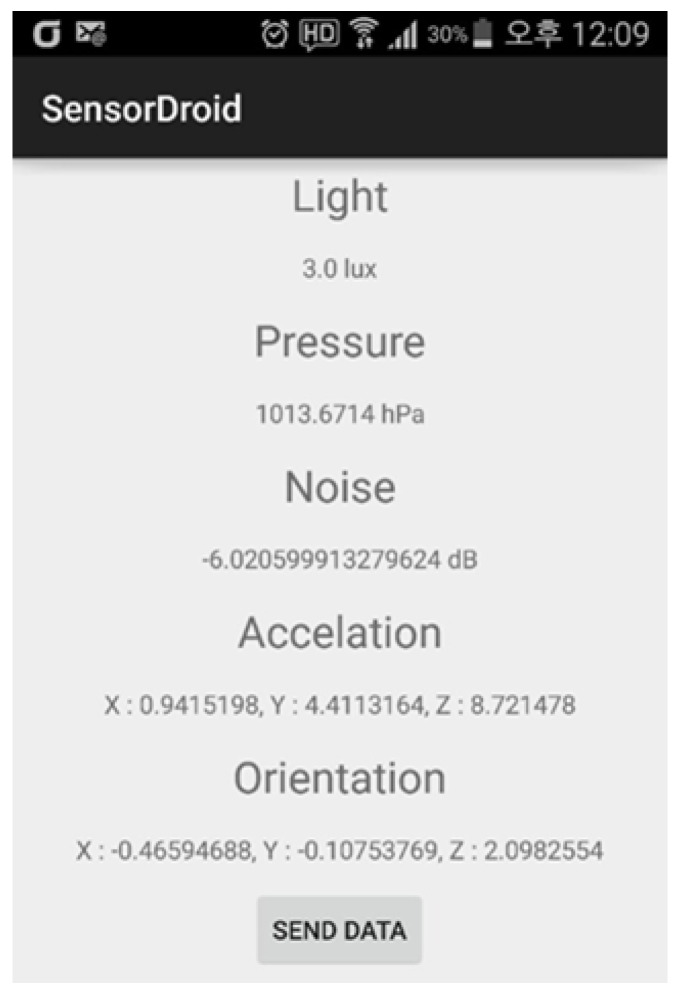
Environment information output result using Android.

Using the sensors of Android-based smartphones, we can collect five pieces of information about the user’s surroundings. A smartphone app and server were developed to acquire environmental information in the vicinity of the user. The program was built using the Android SDK provided by Google and the sensor framework provided by Android. For the smartphone used in the experiment, atmospheric pressure, illuminance, and acceleration can be collected through the sensor manager provided by Android. However, for direction and noise, we had to collect them through other methods because sensors did not exist separately. Direction information was collected by calculating the information of the magnetometer sensor and the acceleration sensor. In addition, noise information was collected by recording sound for a certain period of time using an audio recorder and calculating the size. The server module for connection to the computer was implemented as a JSP server using Java servlets and the Apache Tomcat web container. Figure 3 shows the calculated results through the smartphone.

### 4.2. Facial Expression Recognition, Age and Gender Estimation

The facial information of users, including their expressions, age, and gender, is analyzed in our developed system utilizing two datasets and algorithms for facial detection, age and gender estimation, and expression recognition.

In this study, a Haar-like feature-based algorithm similar to Haar was employed for face detection [21,22]. For facial expression recognition, age estimation, and gender estimation, the VGGNet model architecture was utilized [23,24,25,26,27,28,29]. VGGNet is a versatile model with a simple and easily understandable structure, making it suitable for various applications. It has a deeper architecture compared to many other models, consisting of multiple convolutional layers with small filter sizes, enabling it to capture fine-grained features and learn hierarchical representations. Additionally, the VGGNet architecture is highly adaptable and well-suited for other tasks. For instance, a pre-trained VGGNet model on large-scale image classification datasets like ImageNet can be fine-tuned for facial expression recognition, leveraging the learned representations. VGGNet demonstrates strong performance in various computer vision tasks, including facial expression recognition, by capturing intricate facial features and improving accuracy in recognizing different expressions. Different loss layers were applied based on the data characteristics using the VGG16 architecture for estimation and recognition of each object. The utilization of different loss layers based on the nature of the data further enhances accuracy for different tasks such as expression, age, and gender recognition. For expressions and gender, where the differences between classes are clear, a softmax loss layer was utilized. For age estimation, where the differences between classes are not as distinct, the data was treated as continuous and a regression loss layer, specifically the Euclidean loss layer, was employed to improve accuracy.

**Figure 4:**
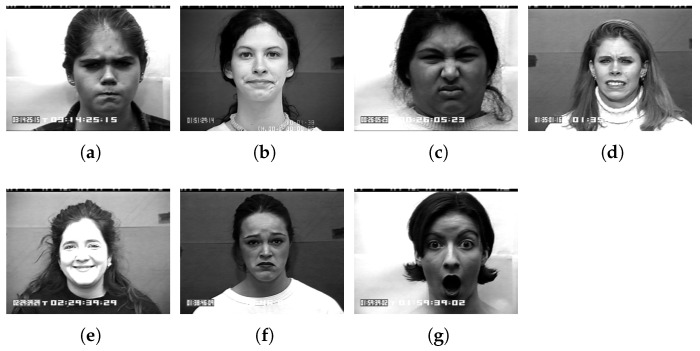
Examples of a label for facial expression recognition: (**a**) anger, (**b**) contempt, (**c**) disgust, (**d**) fear, (**e**) happy, (**f**) sad, (**g**) surprise.

The extended Cohn-Kanade (CK+) dataset was utilized for facial expression recognition [30,31]. This dataset encompasses seven facial expression categories, including anger, disgust, fear, happiness, sadness, surprise, and contempt. It comprises 593 videos captured from 123 individuals aged between 18 and 50 years old (Figure 4).

To evaluate the impact of image pre-processing on accuracy, we drew insights from the study conducted by Lopes et al. [32]. They investigated the influence of image pre-processing operations on the accuracy of facial expression recognition. Lopes et al. achieved impressive results using CNN architecture, with an accuracy of 96.76% for six facial expressions and 95.75% for seven facial expressions. Building upon this previous work, in our study, we focused on evaluating the accuracy of facial expression recognition without performing any specific image pre-processing steps. Instead, we utilized the raw face images and applied a Haar-like feature-based face detector to extract the face region. Subsequently, the resulting images were used as training and validation data.

In this paper, labels of the CK+ dataset were divided into 0 to 7 for facial expression recognition: 0 = Neutral, 1 = Anger, 2 = Contempt, 3 = Disgust, 4 = Fear, 5 = Happy, 6 = Sad, 7 = Surprise. We also extracted and used 1635 frames from the 327 video clips in our dataset. Then, a Haar-like feature-based face detector was used in each frame to cut out only the face region and used as training and validation image data. The experiment used 1330 training data and 305 test data. As a result, an accuracy of about 89% was obtained for eight facial expression classes.

The MORPH database academic version was utilized for age and gender estimation in this study [33]. The MORPH database consists of 55,134 facial image data of 13,618 people, providing the largest amount of personal information among current public face databases.

In age estimation, we utilized the MORPH dataset academic version, which was divided into 44,108 training images and 11,026 testing images. The age range was classified into 45 classes, spanning from 16 to 60 years old. To handle ages below 16, we grouped them into the 16-year-old class, while ages over 60 were classified into the 60-year-old class. This approach allowed us to effectively categorize a wide range of ages. During the age estimation accuracy measurement experiments, we set the error range to ±5 years. This expanded recognition error range was chosen to enhance the recognition success rate and provide greater user convenience. Age estimation is a challenging task, and setting a larger recognition error range, such as ±5 years, significantly improves the probability of achieving higher recognition success rates, leading to improved user satisfaction. Furthermore, it is worth noting that users are often more interested in obtaining approximate age ranges rather than precise ages. Therefore, by expanding the recognition error range to ±5 years, users can easily and simply interpret the recognition results, aligning with their expectations and preferences.

Several studies have been conducted on age and gender estimation using the MORPH dataset. Hiba et al. achieved a Mean Absolute Error (MAE) value of 1.13 using the Hierarchical Attention-based Age Estimation method [34], while Bin et al. achieved an MAE value of 1.969 using the DLDL-v2 method [35]. These studies have served as valuable references for our work.

The experiment revealed that the overall accuracy of age estimation was approximately 84%. As depicted in Table 2, accuracy was highest for teenagers and lowest for individuals in their 60s. The accuracy of age estimation was relatively low for people in their 20s and 30s due to fewer changes in facial features as they age. Moreover, the accuracy was lower for individuals aged 40 and above compared to younger age groups, likely due to the lack of sufficient data for this specific age range.

For gender recognition, a total of 55,134 data were used, which were split into 36,754 training data and 18,380 test data [33]. Among the MORPH data set, which contained 46,640 male data and 4889 female data, 15,192 male data and 3188 female data were used as test data. The overall accuracy of gender estimation was approximately 98%. Table 3 displays the accuracy of gender estimation for each gender.

The datasets used in this study contained overwhelmingly more data from Caucasians and Africans than from Asians. Since most image datasets and deep learning models are trained on Western data, the models are more adept at recognizing Western facial features and may struggle with recognizing East Asian characteristics. Moreover, East Asian faces have distinct features in terms of facial structure, eyes, nose, mouth, etc., which can lead to incorrect results if recognized as Western faces. In particular, East Asian smiles have differences in eye and mouth shape compared to Western smiles, making it challenging to accurately recognize East Asian smiles. Generally, the number of facial muscles used to create facial expressions varies between races, with Asians having relatively fewer muscles for making facial expressions. As a result, the recognition rate may decrease in facial expression recognition and gender estimation for East Asian individuals. Furthermore, since the experiments were conducted with a relatively younger Asian population compared to other races, the users were often perceived as younger than their actual age.

## 5. Image Recommendation System

This study investigates how color psychology, environmental data, and image generation techniques using GAN algorithms can be combined to offer personalized image recommendations that consider an individual’s emotional state and surrounding conditions. The purpose of the study is to explore how understanding the influence of colors on human emotions, incorporating environmental data, and employing image colorization methods can result in customized image suggestions that cater to a person’s specific emotional state and environment.

**Figure 5:**
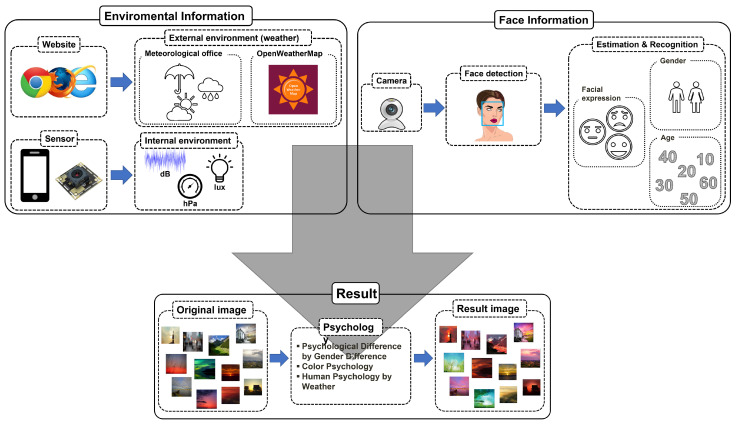
Recommendation system.

Human emotions can be influenced by the weather and the surrounding environment, and they are often expressed through facial expressions. Therefore, when recommending suitable images to a user, it is crucial to consider various factors, including the current external and surrounding environment, the user’s emotions, gender, and age. In this paper, we developed a system (shown in Figure 5) that provides appropriate image information by combining environmental data with the user’s current emotion, age, and gender information.

**Figure 6:**
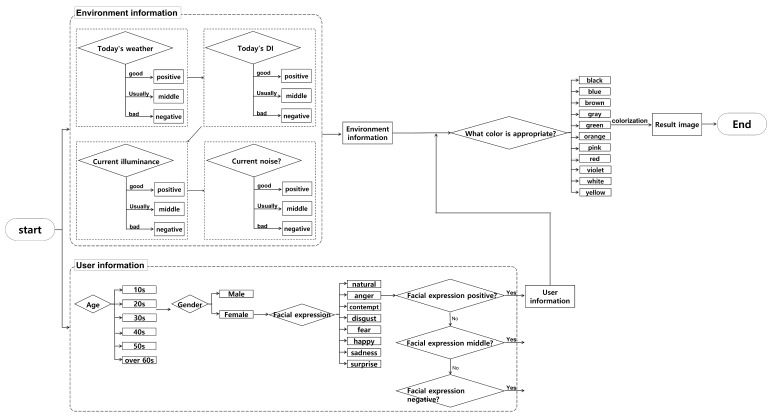
Total flow chart.

This paper proposes a system that analyzes gender-based differences in emotional expressions and visually presents the results using environmental data and color psychology. Our system combines the results of three environmental data categories and emotional expressions based on gender and recognizes age to present the outcome visually, as shown in Figure 6. However, the impact of age on the environment and color psychology remains unclear due to the lack of theoretical research. However, the impact of age on the environment and color psychology remains unclear due to limited theoretical research. Consequently, we plan to incorporate age analysis results into our system once theoretical findings become available. At present, the system presents age recognition results to users solely for the purpose of user satisfaction. Prior to actual system testing, feedback was gathered from experiment participants that they would prefer to appear younger than their actual age. In response to this feedback, our system slightly adjusts the age recognition output to present a younger age group and increase user satisfaction. Specifically, for users over the age of 30, the age group output is adjusted to appear younger than the actual recognition result. In the future, further research will be conducted to enhance the accuracy and reliability of age recognition, thereby improving the overall performance of the system.

In this study, we classified the recognized eight facial expressions into three categories: positive, neutral, and negative. To categorize the expressions, we first distinguished between positive and negative expressions. However, “surprise” proved challenging to categorize strictly as positive or negative. Therefore, we placed “surprise” in the neutral category alongside the “natural” expression. The system utilized three categories of environmental data, as outlined in Table 4. These categories were based on the three facial expression classifications mentioned earlier. The selection of environmental data was designed to align with and complement the facial expression categories, enhancing the overall accuracy and relevance of the system’s recommendations.

First, weather information received through an API was categorized into three types. Although weather perception can be subjective and vary among individuals, we categorized it based on the general consensus of how most people feel about the weather. During the survey conducted for this research, it was observed that some individuals perceived rainy or snowy weather as favorable conditions. However, it was found that this opinion was held by a minority of participants, and the majority tended to perceive other weather conditions as preferable. By considering the overall perception of weather conditions, we aimed to capture the commonly shared viewpoint and provide recommendations based on the majority’s preferences. This approach ensures that the system’s output aligns with the expectations and preferences of a broader user base.

Second, the DI index was divided into three categories. The DI was calculated from the temperature and humidity received through an API. If the DI was over 75%, most people experienced severe discomfort and it was classified as negative. If the DI was between 68-75%, people did not experience severe discomfort but felt some discomfort and were classified as neutral. If the DI was 68% or less, it was classified as positive since everyone feels comfortable at this level. By utilizing the DI index, which integrates temperature and humidity, the system aimed to capture the overall comfort level experienced by individuals in various environmental settings. This information played a crucial role in providing appropriate image recommendations based on factors such as the user’s emotional state, age, and gender.

Lastly, noise and illuminance data received from smartphones were classified into three categories. Noise below 45 dB, which is typical for a residential area, was classified as positive. If the noise level was 60 dB or higher, it was classified as negative since it can negatively affect human psychology. Illuminance was classified as positive when it was bright, neutral when it was moderate, and negative when it was dark. By considering these factors, the system aimed to incorporate the impact of noise and illuminance on the user’s environment and emotional state. This allowed for the provision of image recommendations that aligned with the user’s preferences and the overall atmosphere of their surroundings.

Color psychology investigates how colors can influence human behavior and emotions. Research has established a significant correlation between color and physiological signals. For example, Kaiser et al. (1984) found that color affects both visual and non-visual effects, indicating the impact of color on our perception and experiences [36]. Valdez et al. (1994) identified specific colors as the most and least pleasant, highlighting the subjective nature of color perception [37]. Mehta et al. (2009) demonstrated that different colors can affect cognitive task performance, further emphasizing the influence of color on our cognitive processes [38]. Stone (2001) explored how environmental backgrounds and colors can evoke positive moods, illustrating the emotional impact of colors in our surroundings [39].

Color psychology has found that warm colors generally have a stronger positive effect, while cool colors tend to have a stronger negative effect (Table 5). For example, warm hues such as red, orange, and yellow create a welcoming and friendly atmosphere, eliciting stronger positive responses. These colors are associated with activity, stimulation, and conveying tension and excitement. Middle colors are perceived as neutral and provide a stable state, while cool colors are seen as passive, calming, and conveying relaxation and tranquility. However, it is important to consider that the psychological effects of colors can vary based on context, situation, and personal experiences. Thus, the effects proposed in color psychology should be viewed as general guidelines rather than absolute truths.

It is important to note that individuals perceive and interpret colors differently based on their cultural background and personal experiences, leading to variations in emotional responses. Factors such as ethnicity, region, country, climate, geographical conditions, customs, and religion shape our understanding of colors. Additionally, common associations with colors can also influence the emotions they evoke. For instance, the color yellow may evoke cheerful associations such as sunflowers and sunsets. In light of these findings, this study sought to generate personalized image recommendations that consider emotional states, gender, and environmental influences.

The results presented in Table 6 were derived by considering gender, environmental data, and color psychology. This paper hypothesized that there would be gender differences in emotional expression when experiencing negative emotions. It is also assumed that there is no difference in emotional expression when feeling positive emotions. The categories “positive or neutral emotions” (p/n) and “negative emotions” (n) in the tables were obtained by combining the results of all surrounding environmental data. It is assumed that positive or neutral emotions are largely unaffected by the surrounding environment, and there are no gender-specific differences in responses. However, it is assumed that negative emotions are influenced by the surrounding environment and that there are gender differences in the response. Therefore, the influence of the environment is considered in two parts.

The final result value is obtained by counting the number of positives, neutrals, and negatives. For example, if the subject is angry but the weather is clear, DI and noise levels are low, and the illumination is high, four positives are derived as a result (from Table 4). If the target is male, the system provides an image colored green or white from the p/n line of “Anger” in Table 6. By combining gender, environmental data, and color psychology, the system aims to generate personalized image recommendations that align with the user’s emotional state and surrounding conditions.

**Figure 7:**
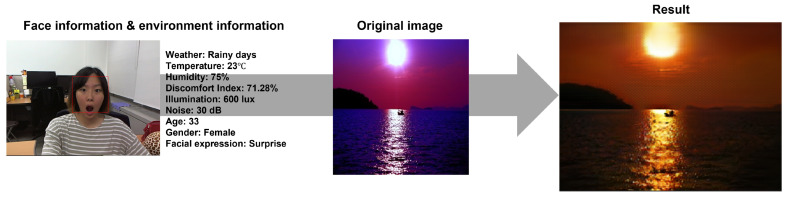
Example result.

**Figure 8:**
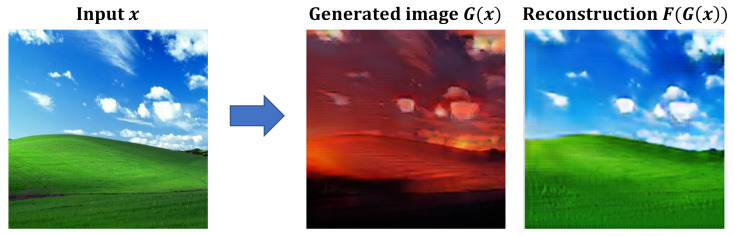
The generated images G(x) and the reconstructed images F(G(x)) from experiments.

In this study, the authors provided images that change color naturally according to color psychology, environmental influences, facial expressions, and gender, focusing on natural landscape images (Figure 7). GAN stands for “Generative Adversarial Network”, which refers to a model where a generator and a discriminator compete with each other to generate data. The generator aims to produce data that resembles real samples, while the discriminator tries to distinguish between the generated data and real data. These constructors and discriminators compete with each other to generate and evaluate images. To do this, they collected 51,802 images from the web using color keywords (black, blue, brown, gray, green, orange, pink, red, purple, white, yellow) and natural keywords (animals, nature, mountains, sky, sea, space, beach, meadow, etc.) and performed color transformation using the CycleGAN algorithm [43,44]. CycleGAN is one of the Generative Adversarial Networks (GAN) algorithms and can learn from an unpaired dataset, eliminating the need to find a correspondence between two images and enabling more extensive image-to-image transformation. CycleGAN is an unsupervised learning-based GAN model for image translation between two domains. Unlike traditional methods, CycleGAN does not require paired data for domain-to-domain transformation. Instead, CycleGAN trains generators and discriminators that are used to map images between the two domains. With these generators and discriminators, CycleGAN performs image transformations from one domain to another. CycleGAN consists of two generators and two discriminators. Each generator is responsible for translating images from one domain to the other. The discriminators determine whether the generated images resemble real images from the corresponding domain. By training these generators and discriminators together, CycleGAN enables image translation between different domains without the need for paired data. Figure 8 shows the color transformation results using CycleGAN.

During the color transformation process of the images, the transformed images were displayed sequentially on the screen in a sequential manner, like a video.

## 6. User Satisfaction Survey

In this study, a survey was conducted to evaluate the performance and effectiveness of an image recommendation system that incorporates environmental and human face information. The questionnaire was designed to gather feedback and insights from the participants and consisted of 31 questions categorized into seven main categories:

- Personal information;
- Environmental information;
- Emotions;
- Test results;
- Satisfaction with the results;
- Scalability;
- Suggestions for improvement.

In the initial section of the questionnaire, personal information was collected from the participants, as presented in Table 7. The purpose of collecting this information was solely to ensure that the participants possessed the professional expertise or relevant background required to understand and evaluate the technology employed in the image recommendation system. By collecting personal information related to the participants’ professional background or expertise, the study aimed to ensure that the feedback and evaluations provided by the participants were informed and meaningful. This approach helps to ensure that the collected data and insights are reliable and reflect the participants’ ability to assess the system based on their knowledge and experience in the relevant field. The personal information collected in this section served as a means to establish the credibility and expertise of the participants in evaluating the technology utilized in the image recommendation system, contributing to the overall validity of the study results.

Table 7 provides an overview of the personal information collected from the participants in the study. The information includes their gender, age, education level, research experience in related fields, and the number of previous experiment participations. The study involved a total of twelve participants, consisting of six males and six females. The age distribution of the participants ranged from those being in their 20s to 40s, representing a diverse range of age groups. In terms of education, the majority of participants held a master’s degree, while some were either Ph.D. candidates or already possessed a Ph.D. degree. This indicates a high level of education and expertise among the participants. Regarding research experience in related fields, most participants had between one and ten years of experience, demonstrating a reasonable level of familiarity with the subject matter. One participant had more than eleven years of research experience, suggesting a significant level of expertise in the field. When considering the participants’ involvement in experiments, the majority had participated in one or two experiments prior to this study, indicating a level of familiarity with experimental procedures. Some participants had even participated in three or more experiments, suggesting a greater level of experience and exposure to research studies. By providing these demographic details, the study aimed to ensure a diverse and knowledgeable participant pool, contributing to the credibility and reliability of the collected data and the subsequent evaluation of the image recommendation system.

In the second item of the questionnaire, participants were asked to provide their opinions on the current weather. This question aimed to assess the participant’s perception of the environmental conditions at the time of the survey. By comparing the responses to this question with the environmental information collected by the system, which was based on data obtained through APIs or other sources, the study sought to determine the consistency between the perceived environmental information reported by the participants and the actual environmental information collected by the system. The findings indicated that there was no significant difference between the environmental information reported by the participants and the environmental information collected by the system. This alignment suggests that the system effectively captured and reflected the real-time environmental conditions experienced by the users, validating the accuracy and reliability of the environmental data used in the image recommendation process. This consistency between the perceived and collected environmental information further strengthens the reliability of the system’s recommendations, as it demonstrates the system’s ability to accurately capture and utilize relevant environmental factors in providing suitable image recommendations to the users.

The third item of the questionnaire aimed to examine the influence of the environment on the participants, as outlined in Table 8. The questionnaire gathered information such as the participants’ preference for specific weather conditions, as well as their perceptions of humidity, light intensity, and noise levels. The objective was to compare the environmental information collected by the system with the users’ own perceptions and preferences. The results indicated that factors such as rain or snow, humidity, and noise had a significant impact on all participants, irrespective of their gender or age. It was observed that the majority of participants did not express a preference for unfavorable weather conditions. Furthermore, when considering humidity, even if the experiment was conducted on the same day, participants’ preferences varied due to differences in their personal perceptions or level of discomfort. This finding highlights the subjective nature of how individuals experience and respond to environmental factors such as humidity.

In the fourth item, we collected opinions on the emotional changes experienced by users after the system test (Table 9, Table 10 and Table 11).

Table 9 shows the responses to the question “Did your mood change positively after the system test?”. Most users answered in the affirmative, indicating that the use of the system had a positive effect on their mood. Table 10 shows the reasons behind this positive change. The majority of users attributed it to the atmosphere of the resulting image. These results suggest that the visual information, such as the color and mood of the image, played a significant role in shaping the user’s emotional response to the system.

Table 11 presents the reasons why some users experienced negative emotional changes after using the system. Users who reported a negative change cited the indoor environment as the primary reason. This was likely due to the hot and humid weather conditions on the day of the system test.

Overall, these findings suggest that the system’s visual output has a significant impact on the emotional response of users, highlighting the importance of paying attention to the quality and atmosphere of the generated images.

Based on the results shown in Table 12, it appears that the users were generally satisfied with the system’s ability to provide appropriate images. Specifically, for items 5.1 and 5.4, which asked about the atmosphere and color of the images matching the surrounding environment and emotional state, respectively, the majority of users rated the system as “Neutral” or better, with several users rating it as “Good”. For item 5.2, which asked about the mood of the images matching the emotional state, the majority of users rated the system as “Good”, while for item 5.3, which asked about the color of the images matching the surrounding environment, the majority of users rated the system as “Neutral” or better, but there were also a significant number of users who rated it as “Bad”. For items 5.5 and 5.6, which asked about age- and gender-appropriate images, respectively, the majority of users rated the system as “Neutral” or better, but there were also some users who rated it as “Bad”. Overall, these results suggest that the system was generally successful in providing appropriate images to users.

In item 6 of the questionnaire, users’ feedback regarding the scalability of our system was obtained (Table 13). The feedback included assessments of the system’s effectiveness when installed outdoors and users’ willingness to continue using the system once it becomes commercially available. Analysis of the responses revealed that installing the system outdoors has a positive impact on individuals. This suggests that users perceive the system as valuable and beneficial in an outdoor setting, further indicating its potential scalability and applicability in real-world environments. Additionally, the majority of users expressed their willingness to continue using the system once it is commercialized. This favorable response indicates a high level of user satisfaction and interest in the system’s capabilities and functionalities. However, it is worth noting that some users provided feedback regarding the resolution of the resulting images. This issue is acknowledged and attributed to the use of CycleGAN, which was employed for image colorization in the system. These improvements aim to ensure superior quality and visually appealing results, thereby augmenting user satisfaction.

In conclusion, our system exhibits promising potential for scalability and applicability in various settings, encompassing both indoor and outdoor spaces. We will continue to work on improving the system to meet the needs of different users and environments.

## 7. Conclusions

In this study, a recommendation system was developed that suggests suitable images for users based on various factors, including their emotions, gender, age, and surrounding environment. Facial expression, age, and gender information were collected using the CK+ and MORPH datasets, with a CNN algorithm employing the VGG16 model architecture. Additionally, environmental data were collected using smartphone sensors and two APIs.

To provide customized images, we combined the facial and environmental data with color psychology and transformed the collected images using the CycleGAN algorithm. The system displayed the transformed images sequentially on the screen, like a video.

Our system provides customized color images that match the user’s current psychological state, combining color psychology with environmental influences and facial expressions. The performance of our system was evaluated based on facial expression recognition accuracy, age estimation accuracy, and gender estimation accuracy, all of which demonstrated high levels of accuracy. Additionally, positive feedback from participants in the user satisfaction survey indicated that the system effectively delivered personalized emotional services that stimulated users’ visual pleasure.

Emotion recognition technology, as demonstrated by our system, has the potential to reduce errors in emotional communication between humans and computers and efficiently provide users with desired information or services. Furthermore, in marketing, emotion recognition can contribute to establishing tailored marketing strategies that align with consumers’ needs. The provision of personalized emotional services is expected to have a positive impact on users.

In conclusion, our study has demonstrated the potential to enhance user experiences by providing personalized image recommendations that consider users’ emotions, gender, age, and surrounding environment. Our system offers advantages in accurately estimating and recognizing human emotions, age, and gender in real-time, as well as incorporating environmental influences and color psychology to create customized color images. The positive evaluation results and user feedback support the effectiveness and user-friendliness of our system.

The system captures facial expressions to estimate emotions, but it may not fully capture the complex emotional experiences that are not solely expressed through facial expressions. In future research, these limitations will be addressed by integrating additional factors such as speech and speech patterns to improve the system’s performance and applicability in various fields. Furthermore, we will strive to overcome the identified limitations by collecting more high-quality data and researching additional emotion classification algorithms, aiming to enhance the system’s performance and usability in diverse domains.

## Figures and Tables

**Table 1:** Light level.

Outdoor	Illuminance (lux)	Indoor
Sunlight	107,527	
Full daylight	10,752	
Overcast day	1075	
	500	Office, Kitchens
	300	Conference room, Dining room
	200	Lobbies, Atria, Living room
	150	Auditorium, Dining
Very dark day	107	
	100	Restroom
	50	Garage
Twilight	10.8	
Deep twilight	1.08	
Full moon	0.108	
Quarter moon	0.0108	
Starlight	0.0011	
Overcast Night	0.0001	

**Table 2:** Age-specific accuracy.

Age	Data (Total: 11,026)	Accuracy
<20	1493	0.96
20∼29	3266	0.88
30∼39	3071	0.83
40∼49	2409	0.80
50∼59	720	0.70
60<	67	0.55

**Table 3:** Gender estimation accuracy.

Gender	Data (Total: 18,380)	Accuracy
Male	15,192	0.96
Female	3188	0.99

**Table 4:** Three categories of environmental data.

	Weather	DI (%)	Noise (dB)	Illuminance (lux)
Positive	Clear Partly Cloudy	75∼100	<45	1000
Neutral	Mostly Cloudy Cloudy Rain/Snow (<1 mm)	68∼75	46∼59	110∼1000
Negative	Rain Snow	0∼68	60<	<110

**Table 5:** Effects of colors [40,41,42].

Color	Psychological
Red	Warm color, warmth, positive, passion, anger, vitality, power, happiness, pleasure, struggle
Pink	Warm color, softness, kind, sophistication, sincerity, feminine
Orange	Warm color, pleasure, confidence
Yellow	Warm color, happy, smooth, gaiety
Brown	Warm color, equality, stability, firm, comfortable, cozy
Green	Middle color, hope, balance, harmony, neutrality, concentration, friendly, stability
Blue	Cold color, a symbol of absolute rest, trust, fidelity, stability, propulsion, sadness, anguish
Violet	Cold color, noble, authority, sophistication, power, secret, sadness, mature
White	Achromatic color, purity, peace, happiness, sincerity, vitality, optimism
Gray	Achromatic color, neutrality, compromise, gentle, moderate, gloom
Black	Achromatic color, sophistication, highest negative, grief, fear, abstinence, serious, strict, exclusion

**Table 6:** Emotions and colors by gender.

Gender	Color	Neutral	Happy	Surprise	Anger		Contempt		Disgust		Fear		Sadness	
					p/n	n	p/n	n	p/n	n	p/n	n	p/n	n
Female	Red	o	o	o										
	Pink	o	o	o										
	Orange	o	o	o										
	Yellow	o	o	o										
	Green	o	o	o										
	Blue	o	o	o										
	Violet		o											o
	White	o	o	o										
	Gray				o	o	o	o	o		o		o	
	Black									o		o		o
	Brown				o	o	o	o	o		o			
Male	Red	o	o	o										
	Pink	o	o	o							o		o	
	Orange	o	o	o									o	
	Yellow	o	o	o					o					
	Green	o	o	o	o		o		o		o		o	
	Blue	o	o	o		o		o		o		o		o
	Violet		o											
	White	o	o	o	o		o		o		o			o
	Gray					o						o		
	Black					o						o		
	Brown													

**Table 7:** Subject’s personal information and questionnaire results.

Questions		Number of People	
1.1. Gender		Male: 6	Female: 6
1.2. Age	10s	0	
	20s	3	3
	30s	2	3
	40s	1	
	50s	0	
1.3. Education	Master’s course	3	3
	M.S		
	Ph.D. student	1	1
	Ph.D candidate	2	1
	Ph.D		1
1.4. Research experience in related fields of research (year)	<1 year	1	3
	1∼3	3	
	4∼6		1
	7∼10	2	2
	11<		
1.5. Number of experiment participation	1	3	4
	2	3	2
	3<		

**Table 8:** Factors affecting emotions and their strength of influence.

Factors Affecting	Weak	⇐	Neutral	⇒	Strong
Weather	1	2	2	7	0
Rain/Snow	1	0	2	7	2
Humidity	0	1	1	6	4
Noise	0	0	4	5	3
Illumination	0	2	6	4	0

**Table 9:** Positive mood change after system test by gender and age group.

	20s		30s		40s
	Male	Female	Male	Female	Male
Strongly Disagree					
Disagree			1		
Neutral	2	1			
Agree	1	2	1	3	1
Strongly Agree					

**Table 10:** Reasons for positive mood change after system test, by gender and age group.

	20s		30s		40s
	Male	Female	Male	Female	Male
Contents of the resulting image					
Color of the image				2	1
The atmosphere of the image	2	2	1	1	
No change	1	1	1		
Other factors					

**Table 11:** Reasons for negative mood change after system test.

	20s		30s		40s
	Male	Female	Male	Female	Male
Image inappropriateness	1			1	
Weather					
Indoor environment			1		
No change	2	3	1	2	1
Other factors					

**Table 12:** Satisfaction with the results of using the system.

		Bad	⇐	Neutral	⇒	Good
5.1.	The atmosphere of the image that matches the surrounding environment			1	3	2
5.2.	The mood of the image that matches the emotional state				5	1
5.3.	The color of the image that matches the surrounding environment			2	4	
5.4.	The color of the image suitable for the emotional state			1	3	2
5.5.	Age-appropriate images			1	5	
5.6.	Gender-appropriate images				6	

**Table 13:** Scalability of the system.

		Bad	⇐	Neutral	⇒	Good
6.1.	Do you think it will be helpful when it is installed outside?	1		9		2
6.2.	Are you willing to continue using this system when it was installed?	4		7		1

## Data Availability

The data are not publicly available due to restrictions, e.g., privacy or ethical.
